# P-193. Multiplex testing optimization for Field Work Pandemic Prevention in West Africa

**DOI:** 10.1093/ofid/ofaf695.416

**Published:** 2026-01-11

**Authors:** Liam Stenson, Elyse Stachler

**Affiliations:** Broad Institute of MIT and Harvard, Cambridge, MA; Broad Institute of MIT and Harvard, Cambridge, MA

## Abstract

**Background:**

Diagnostics have long faced challenges—including contamination, limited specificity toward pathogens, and inefficiencies in workflow—when working to identify the diseases affecting populations in diverse global communities. These challenges are amplified in an increasingly interconnected world where international travel and trade accelerate pathogen spread. The Combinatorial Arrayed Reactions for Multiplexed Evaluation of Nucleic acids (CARMEN) addresses some of these problems by combining the sensitivity of RT-PCR with the specificity of CRISPR-Cas13-based detection to enable high-throughput and accurate identification of rapidly evolving pathogens.

**Figure:**
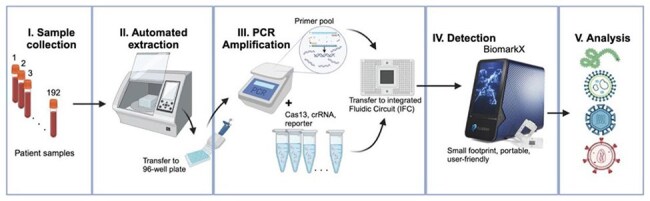
Robust detection of the respiratory virus panel using the CARMEN-BiomarkX Figure 1A. from our preprint: Multiplexed detection of febrile infections using CARMEN M. Kamariza, K. McMahon, L. Kim, N.L. Welch, L. Stenson, L. Allan-Blitz, G. Sanders, P. Eromon, A.M. Iluoreh, A. Sijuwola, O.O. Ope-ewe, A.O. Ayinla, C. l’Anson, I. Baudi, M.F. Paye, C. Wilkason, J. Lemieux, A. Ozonoff, E. Stachler, C.T. Happi, P.C. Sabeti medRxiv 2024.07.15.24310364; doi: https://doi.org/10.1101/2024.07.15.24310364

**Methods:**

Previously, we implemented CARMEN in collaboration with our partners in West Africa, testing respiratory and bloodborne panels across up to 17 pathogens in 190 patient samples. To improve ongoing surveillance of emerging pathogens, our team has prioritized design optimization to improve specificity, enhance capture of viral strain diversity, and ensure that workflows are well suited for deployment at partner sites in Lower-Middle-Income Countries (LMICs), where implementation barriers are often greater.

**Results:**

The current optimization includes a redesigned configuration and expands assay panels to detect both DNA and RNA pathogens relevant to the region, such as Malaria, mpox, O’nyong’nyong virus, and Hepatitis B. We also reduced PCR cycles to minimize contamination and tested the stability of our Combined Positive Control (CPC) at room temperature for long-term transport and storage. Additionally, we conducted temperature simulation experiments to assess nucleic acid degradation over time.

**Conclusion:**

These improvements support pandemic preparedness by enabling timely, reliable diagnostics for current and emerging pathogens while making the platform more accessible and practical for LMICs.

**Disclosures:**

All Authors: No reported disclosures

